# Characterization of Viral Communities of Biting Midges and Identification of Novel *Thogotovirus* Species and *Rhabdovirus* Genus

**DOI:** 10.3390/v8030077

**Published:** 2016-03-11

**Authors:** Sarah Temmam, Sonia Monteil-Bouchard, Catherine Robert, Jean-Pierre Baudoin, Masse Sambou, Maxence Aubadie-Ladrix, Noémie Labas, Didier Raoult, Oleg Mediannikov, Christelle Desnues

**Affiliations:** 1 Unité de Recherche sur les Maladies Infectieuses et Tropicales Emergentes (URMITE), UM63 CNRS 7278 IRD 198 INSERM U1095, Aix-Marseille Université, Marseille 13005, France; sarah.temmam@gmail.com (S.T.); sonia.monteil@univ-amu.fr (S.M.-B.); catherine.robert@univ-amu.fr (C.R.); tem.timone@gmail.com (J.-P.B.); massezorro1@gmail.com (M.S.); lamenacexr@yahoo.fr (M.A.-L.); labas-noemie@live.fr (N.L.); didier.raoult@gmail.com (D.R.); olegusss1@gmail.com (O.M.).; 2 Fondation IHU Méditerranée Infection, Pôle des Maladies Infectieuses et Tropicales Clinique et Biologique, Fédération de Bactériologie-Hygiène-Virologie, Centre Hospitalo-Universitaire Timone, Méditerranée Infection, Assistance Publique–Hôpitaux de Marseille, Marseille 13005, France

**Keywords:** viral metagenomics, biting midges, zoonoses, epizooties, thogotovirus, rhabdovirus

## Abstract

More than two thirds of emerging viruses are of zoonotic origin, and among them RNA viruses represent the majority. *Ceratopogonidae* (genus *Culicoides*) are well-known vectors of several viruses responsible for epizooties (bluetongue, epizootic haemorrhagic disease, *etc.*). They are also vectors of the only known virus infecting humans: the Oropouche virus. Female midges usually feed on a variety of hosts, leading to possible transmission of emerging viruses from animals to humans. In this context, we report here the analysis of RNA viral communities of Senegalese biting midges using next-generation sequencing techniques as a preliminary step toward the identification of potential viral biohazards. Sequencing of the RNA virome of three pools of *Culicoides* revealed the presence of a significant diversity of viruses infecting plants, insects and mammals. Several novel viruses were detected, including a novel *Thogotovirus* species, related but genetically distant from previously described tick-borne thogotoviruses. Novel rhabdoviruses were also detected, possibly constituting a novel *Rhabdoviridae* genus, and putatively restricted to insects. Sequences related to the major viruses transmitted by *Culicoides*, *i.e.*, African horse sickness, bluetongue and epizootic haemorrhagic disease viruses were also detected. This study highlights the interest in monitoring the emergence and circulation of zoonoses and epizooties using their arthropod vectors.

## 1. Introduction

There are more than 200 viral species that are known to be able to infect humans. Since the discovery of the yellow fever virus in 1901, three to four new species have been discovered every year [1]. There is, however, a substantial pool of unknown human viral species which are yet to be discovered, and the development and democratisation of Next-Generation Sequencing techniques (NGS) has enabled the identification of many new viruses, for which the potential risk to humans remains mostly unknown. More than two-thirds of viral species infecting humans are of zoonotic origin, and RNA viruses represent more than 70% of these [1,2], resulting in the recent increase in studies of viral communities of wild and domestic animals [3]. However, and despite the fact that haematophagous arthropods usually act as vectors of transmission between animals and humans, few studies have analysed viral communities of arthropods [3]. The studies that have been previously conducted have focused on mosquito viromes [4,5,6,7,8] and have reported the discovery of novel viruses, including bunyaviruses, rhabdoviruses, reoviruses, and flaviviruses. More recently, two studies described the composition of viral communities of hard ticks [9,10] and reported the identification of novel viruses belonging to the *Nairovirus*, *Phlebovirus*, and *Flavivirus* genera, highlighting, as for mosquitoes, potential new zoonotic risks to humans.

*Ceratopogonidae*, and particularly the genus *Culicoides*, are small (1–3 mm) and highly diverse midges, with more than 1300 species around the world [11,12]. Of these, 96% are haematophagous and only the females require blood meal for egg fertilisation. Biting midges are well-known vectors of several parasites (such as *Mansonella sp*.) [13,14] and viruses infecting animals (*i.e.*, bluetongue virus, African horse sickness virus, epizootic haemorrhagic disease virus, Schmallenberg virus, *etc.*) [15]. The Oropouche virus is the only human virus known to be transmitted by biting midges in Latin and South America [16].

We report here the first comprehensive analysis of viral communities from Senegalese *Culicoides* biting midges and the identification of several novel viruses, including a novel thogotovirus and a novel rhabdovirus.

## 2. Materials and Methods

### 2.1. Sample Collection

Biting midges were collected using a modified Centers for Disease Control (CDC) light trap in the villages of Dielmo and Ndiop in the Sine-Saloum region of Senegal, in November 2013. Traps were placed near places where cattle rested and were left overnight. Morphological identification of the arthropods was conducted the following morning. Three types of pools of arthropods were created: STE0043 (more than 200 adult *Culicoides* sp., with no distinction between male and female, or engorged status); STE0044 (N = 15 engorged female *Culicoides imicola*) and STE0045 (N = 100 non-engorged male and female *Culicoides imicola*).

### 2.2. Virome Preparation

The three pools of arthropods were crushed with two 3 mm tungsten beads and a TissueLyser at 25 Hz for two minutes (Qiagen, Courtaboeuf, France). The clarified supernatant was subsequently used as a template for virome preparation, as previously described [17]. Briefly, the clarified supernatant was filtered through a 0.45 µm filter (Millipore, Molsheim, France), and free nucleic acids were digested with a cocktail of nucleases. Finally, the digested supernatant was purified onto a discontinuous 66%–30% sucrose gradient and ultracentrifuged at 130,000 g for two hours at + 4 °C on a MLS-50 rotor (Beckman-Coulter, Villepinte, France). The viral fraction was harvested at the interphase between the 66% and 30% sucrose layers. Total RNAs were extracted from the purified viral fraction with Trizol LS^®^ reagent (Life Technologies, Saint Aubin, France), according to the manufacturer’s recommendations. Random amplification was performed using the Froussard [18] random RT-PCR. and amplification products were purified with Agencourt AMPure Beads (Beckman-Coulter, Villepinte, France) according to the manufacturer’s protocol, eluted to a final volume of 15 µL and sequenced using MiSeq Technology using paired-end and barcode strategies according to a Nextera XT library kit in a 2 × 300 bp format (Illumina Inc., San Diego, CA, USA).

### 2.3. Bioinformatic Analyses of Viromes

Raw reads were imported in pairs into the CLC Genomics Workbench 6.0.1 programme (CLC Bio, Aarhus, Denmark) and trimmed according to their quality score, the presence of ambiguities, and their length (reads which were shorter than 50 nt were discarded). The pre-processed viral metagenomes are publicly available on the Metavir server [19] under the “Arthrovirome” project and on the MG-RAST server [20,21] with the identifiers 4604249.3, 4604250.3, and 4604251.3 for the STE0043, STE0044 and STE0045 RNA viromes, respectively.

Cleaned paired reads were assembled into contigs using the CLC Genomics programme and the following parameters: word size of 20 nt, minimum contig length of 200 nt, mismatch cost of 2, insertion/deletion cost of 3, length fraction of 0.5 and similarity fraction of 0.8. Contigs and non-assembled reads were compared to the NCBI nucleotide database using the BlastN algorithm, with a minimum coverage of 50%, minimum identity of 50% and E-value < 10^−5^. Sequences having no significant hits according to the criteria described above were classified as “unknown”. Contigs were then compared to the NCBI viral database using the BlastX program with a minimum coverage of 50%, minimum identity of 50% and E-value < 10^−5^. Finally, to confirm the specificity of the BlastX result, contigs were compared to the NCBI non-redundant nucleotide database using the same criteria. The taxonomic assignation of contigs was conducted by selecting the best BlastX score result between the two Blast run for each contig. Figure S1 presents the pipeline for bioinformatic analyses.

Principal Component Analysis (PCA) was used to compare data in the MG-RAST server [21] with a maximum E-value of 10^−5^, a minimum identity of 60%, and a minimum alignment length of 15 amino-acids for protein and 15 bp for RNA databases. Data were normalised to values between 0 and 1, and distances were measured using the Bray-Curtis distance matrix.

### 2.4. Phylogenetic Analyses

Contigs with a significant hit for viruses were translated, and predicted open reading frames (ORFs) were aligned with other amino-acid sequences retrieved from the GenBank database using MUSCLE aligner [22] implemented through MEGA6 [23]. The amino-acid substitutions models that best fitted the data were performed on MEGA6 and were considered for all phylogenetic analyses. The best substitution model was selected using the corrected Akaike information criterion. Phylogenetic trees were constructed using Maximum Likelihood (ML) implemented through the MEGA6 package software, according to the selected substitution model. Nodal support was evaluated using 1000 bootstrap replicates. Bayesian phylogenetic inference (BI) was carried out using MrBayes [24] with two independent runs of four incrementally-heated, Metropolis-coupled Markov chain Monte Carlo (MCMC) starting from a random tree. The MCMC were run for 10^6^ iterations and associated model parameters were sampled every 500 generations. The initial 2000 trees in each run were discarded as burning samples and the harmonic mean of the likelihood was calculated by combining the two independent runs.

Molecular evolutionary distances between sequences were calculated using MEGA6 [23]. For analysis of evolutionary distances between thogotoviruses, individual sequences available in GenBank and the *p*-distances algorithm were used. For analysis of molecular evolutionary distances between rhabdoviruses, sequences available in GenBank were grouped according to their recognised or putative genus (defined by phylogenetic analyses) and distances were calculated (i) within genera using the *p*-distance algorithm (ii) between genera using net distance calculations (*i.e.*, MEGA6 takes into account the mean distance within genera) and the *p*-distance algorithm.

### 2.5. Transmission Electron Microscopy (TEM)

Approximately 50 mg of STE0043 arthropod samples were washed in 70% ethanol and crushed in 2 mL of sterile EMEM medium (Life Technologies). The supernatant was harvested after low speed clarification and subsequently filtered through a 0.8-µm filter (Millipore) followed by ultracentrifugation onto a discontinuous 66%–30% sucrose gradient at 130,000 g for two hours at + 4 °C. The viral fraction was harvested at the interphase between the 66% and 30% sucrose layers and fixed for one hour at + 4 °C with 2% final glutaraldehyde. The fixed viral fraction was then diluted to a final volume of 4 mL in PBS and directly adsorbed onto formvar carbon films on 400 mesh nickel grids (FCF400-Ni, EMS) by ultracentrifugation at 130,000 g for one hour at + 4 °C, as previously described [25]. Grids were stained for 10 seconds with 1% molybdate solution in filtered water at room temperature. Electron micrographs were obtained on a Tecnai G2 transmission electron microscope (FEI) operated at 200 keV equipped with a 4096 × 4096 pixel resolution Eagle camera (FEI).

## 3. Results

### 3.1. Diversity of Viral Communities in Haematophagous Biting Midges

RNA viromes of samples STE0043, STE0044 and STE0045 were sequenced using Illumina MiSeq technology. Sequencing statistics are presented in Table 1.

**Figure 1:**
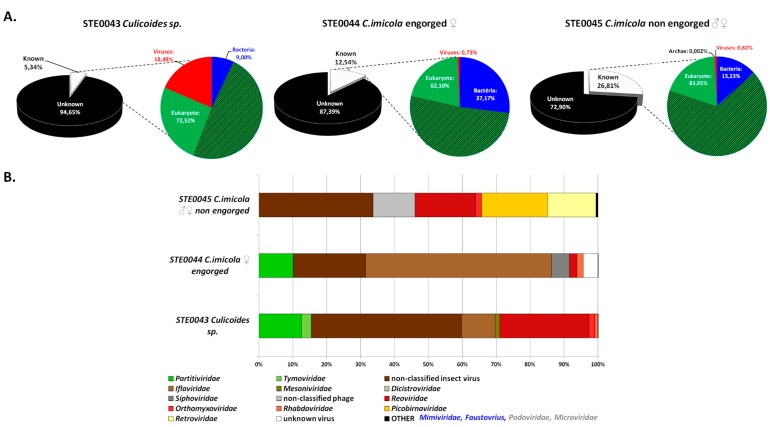
Taxonomic assignment of reads (**A**) BlastN search against the National Center for Biotechnology Information (NCBI) nucleotide database (dashes correspond to the arthropod-borne proportion of eukaryotic reads) (**B**) Relative abundance of viral families in biting midge metagenomes according to their target hosts (Green: plant viruses, Brown: insect viruses, Grey: bacteriophages, Red: arboviruses, Yellow: mammalian viruses, Blue: amoeba-infecting giant viruses).

The taxonomic assignment of reads identified only 5%–25% of sequences which had similarities with known sequences (Figure 1A). Of these, eukaryotes represented the majority of sequences, with 72.52%, 62.10% and 83.95% of total known reads of the STE0043, STE0044 and STE0045 RNA viromes, respectively (Figure 1A). Most eukaryotic reads were assigned to arthropods (>60% of total eukaryotic reads), and they mainly consisted of arthropod ribosomal RNAs. Bacteria-related sequences ranged from 9% to 37% depending on the sample (Figure 1A).

Virus-related sequences represented 0.73%–18.48% of total known reads. Of them, plant viruses (*i.e.*, *Partitiviridae*, *Tymoviridae*) composed 15.48%, 10.10% and 0.00% of total viral reads for STE0043, STE0044 and STE0045 RNA viromes, respectively (Figure 1B). Insect viruses (*i.e.*, *Iflaviridae*, *Mesoniviridae*, *Dicistroviridae*, and non-classified insect viruses) represented the majority of viral reads, with 55.51%, 76.23% and 33.66% of total viral reads for STE0043, STE0044 and STE0045 RNA viromes, respectively. Several mammalian viruses were detected, such as *Picobirnaviridae*-related viruses, but only in the STE0045 *C. imicola* male and non-engorged female virome, with a global abundance of 33.73% of total viral reads. Animal-infecting arboviruses belonging to the *Reoviridae* family were identified and represented 26.34%, 0.04% and 17.79% of total viral reads for STE0043, STE0044 and STE0045 RNA viromes, respectively (Figure 1B). Finally, several reads were assigned to *Orthomyxoviridae* (1.69% and 1.84% of total viral reads for STE0043 and STE0045 viromes, respectively) and *Rhabdoviridae* (0.96% and 1.86% of total viral reads for STE0043 and STE0044 viromes, respectively) but they presented a relatively low identity of 57%–62% in the RNA polymerase after BlastX analysis (Figure 1B). Few DNA viruses were also identified in the RNA viromes (bacteriophages and amoeba-infecting giant viruses, representing 0.02%, 5.24% and 12.97% of total viral reads for STE0043, STE0044 and STE0045 RNA viromes, respectively), possibly due to residual contamination of the RNA fraction by viral DNA (Figure 1B).

**Figure 2:**
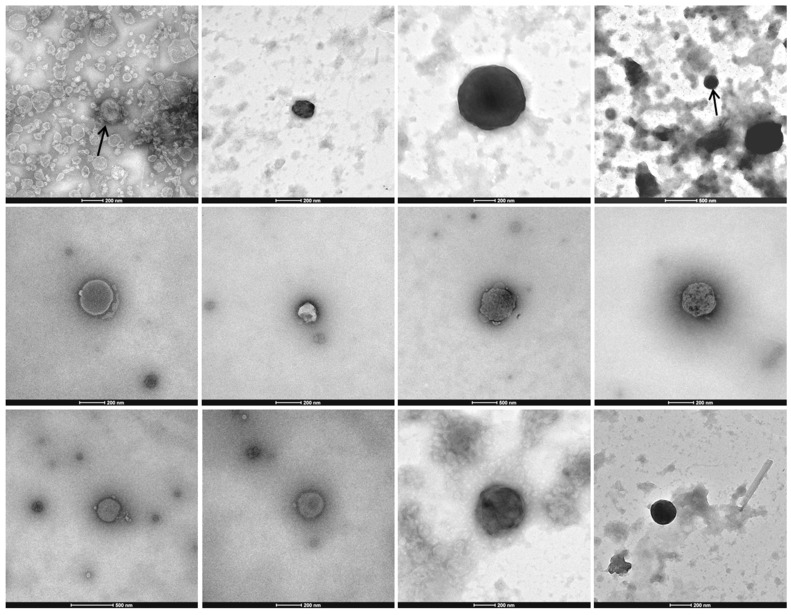
Repertory of transmission electron microscopy images of *Culicoides sp.* viral communities.

Electron microscopy images of the STE0043 *Culicoides sp*. purified viral fraction showed the presence of virus-like particles (VLPs) with various diameters, morphologies, and contrasts (Figure 2). Some VLPs presented a round structure with a distinct envelope, while others appeared with more contrast. The diameters of the particles ranged from 100 nm to 600 nm (Figure 2).

**Figure 3:**
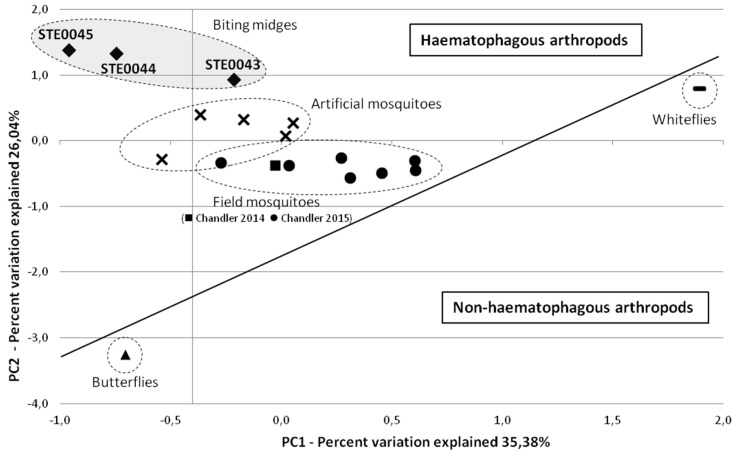
Comparison between viromes of biting midges with available arthropod RNA metagenomes based on a taxonomic classification of reads. Principal component analysis (PCA) was used to compare data in MG-RAST server [21] with a maximum E-value of 10^−5^, a minimum identity of 60%, and a minimum alignment length of 15 amino-acids for protein and 15 bp for RNA databases. Data were normalised to values between 0 and 1 and distances were measured using the Bray-Curtis distance matrix.

Principal component analysis (PCA) was used to compare viral communities of biting midges with other haematophagous and non-haematophagous arthropod RNA viromes available in public databases (Figure 3, Table S1). RNA viromes of biting midges clustered together, but the STE0043 *Culicoides sp*. virome was more distant than the STE0044 *C. imicola* engorged female and STE0045 *C. imicola* male and non-engorged female viromes. In addition, biting midge viromes were closer to field and artificially-infected mosquito metagenomes than to whitefly and butterfly viromes (Figure 3).

### 3.2. Orbiviruses Were Abundant in Senegalese Biting Midges

Within the viral reads, *Reoviridae*-related sequences represented 26.34%, 0.04% and 17.79% in STE0043, STE0044 and STE0045 RNA viromes, respectively; with the presence of bluetongue-related sequences in STE0043 (N = 3656 reads) and STE0045 (N = 678 reads) viromes while epizootic haemorrhagic disease virus (EHDV) was detected in STE0043 (N = 5454 reads) and STE0044 (N = 5 reads) viromes. African horse sickness virus (AHSV) was only detected in the STE0043 *Culicoides sp*. RNA virome (N = 1647 reads).

Various segments of these 10-segmented double-stranded RNA (dsRNA) orbiviruses were detected in the metagenomes. For example EHDV-related sequences matched with VP4 protein of segment 4 in the STE0044 *C. imicola* engorged female virome. In the STE0045 sample, all reads matched with segment 8 (NS2 protein) of the bluetongue virus (BTV). Within the STE0043 *Culicoides sp*. virome, sequences related to segments 1-2-3-4-6-7-8 and 9 of AHSV were present, while NS1 (segment 5) and NS3 (segment 10) were not detected. Segments 1-2-3-4-8 and 9 of BTV and segments 1-3-4-6 and 8 of EHDV were detected, with a global coverage of the genome estimated after mapping at 37.27%, 34.58% and 33.16% for AHSV, BTV and EHDV, respectively, in the STE0043 metagenome (data not shown).

### 3.3. Novel Thogotovirus Species

**Figure 4:**
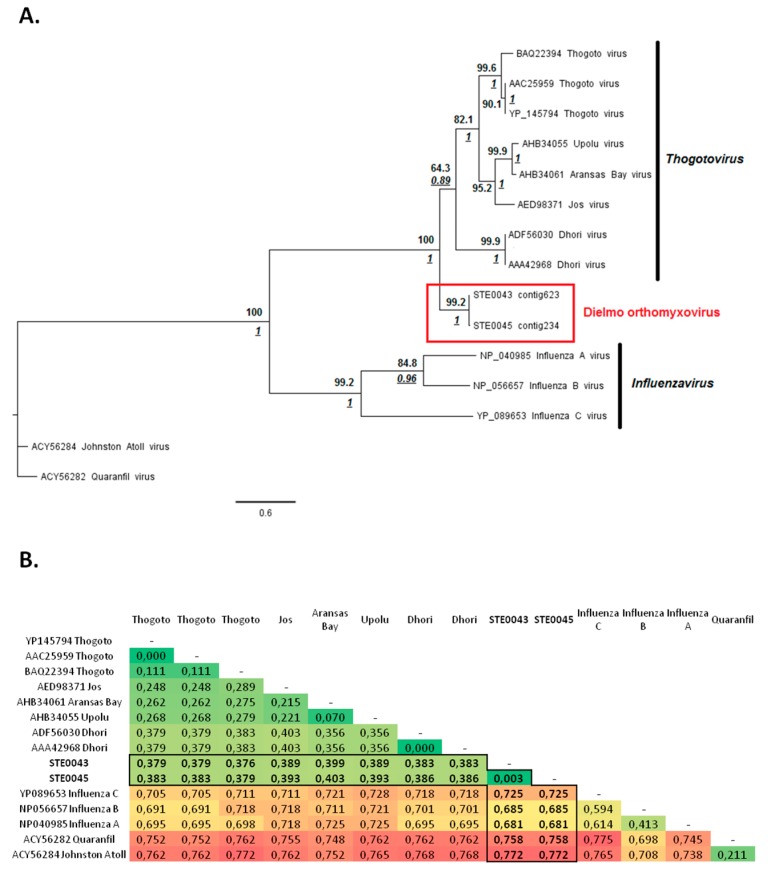
Phylogenetic analyses of Dielmo orthomyxovirus compared to other *Thogotovirus* viruses. (**A**) Phylogenetic analysis of a fragment of 358 amino-acids of PB1. ML analysis was used to fix tree topology. ML analysis was performed on 1000 iterations and bootstrap values are represented in bold. Bayesian posterior probabilities are underlined and represented in italics where nodes coincided with ML. Substitutions models for ML and Bayesian analyses were determined as LG+I+G and rtREV+I+G, respectively. Scale bar indicates the number of amino-acid substitutions per site; (**B**) Matrix of genetic distances observed between PB1 amino-acid sequences of Dielmo orthomyxovirus and other representative thogotoviruses. Diversity was calculated by the pairwise-distance algorithm implemented through MEGA [23].

Within the virome of the STE0043 *Culicoides sp*. and STE0045 *C. imicola* male and non-engorged female samples, large contigs of 1903 nt and 1217 nt, respectively, matched with the viral RNA polymerase PB1 segment of viruses belonging to the genus *Thogotovirus* (family *Orthomyxoviridae*), with a nucleotide identity of 61.26% and 57.61%, respectively. Phylogenetic analyses enabled the identification of a clade formed by the identified thogotovirus-like orthomyxovirus, tentatively named “Dielmo orthomyxovirus” (DOV), with a high bootstrap value of 99.2 and a high posterior probability of 1 (Figure 4A). The clade formed by DOV, placed at the root of the group formed by viruses belonging to the *Thogotovirus* genus, is supported by high bootstrap value and posterior probability, suggesting that DOV could constitute either a novel species within the *Thogotovirus* genus or a novel genus within the *Orthomyxoviridae* family (Figure 4A). However, analyses of genetic distances between DOV and other orthomyxoviruses supported the classification of DOV among the *Thogotovirus* genus rather than a new genus since it presented similar distances with other thogotoviruses and distances in the same range as those observed between other thogotoviruses and *Influenzavirus* genus (Figure 4B).

### 3.4. Novel Rhabdoviridae Genus

Within the virome of the STE0043 *Culicoides sp*. and STE0044 *C. imicola* engorged female samples, large contigs of 1397 nt and 1572 nt, respectively, matched with the viral RNA polymerase of North Creek virus (NCV), a novel rhabdovirus detected in Australian mosquito metagenomes [26]. The new Senegalese rhabdovirus, tentatively named “Dielmo rhabdovirus” (DRV), was distant from North Creek virus, with only 62.61% and 61.06% of nucleotide homologies, respectively. Nucleotide and amino-acid sequences of STE0043 and STE0044 Dielmo rhabdovirus were 100% identical, while they presented a genetic distance from Australian mosquito North Creek virus of 0.352 and 0.377 in nucleotide and amino-acid sequences, respectively.

**Figure 5:**
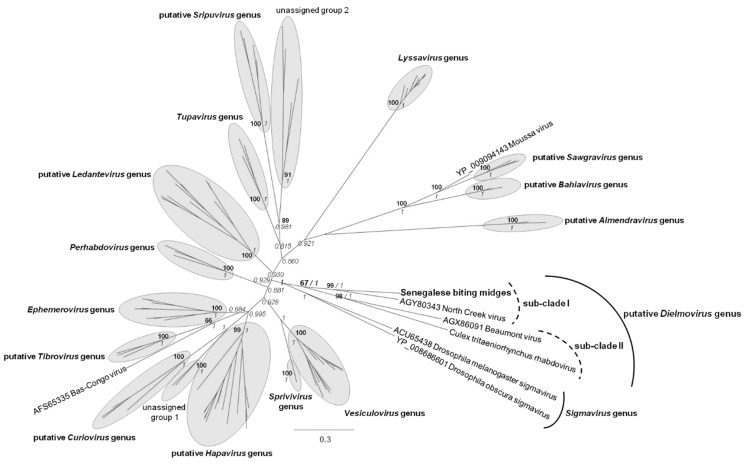
Phylogenetic analysis of *Dielmovirus* genus compared to other *Rhabdoviridae*. Phylogenetic analysis of a fragment of 463 amino-acids of the RNA-dependant RNA polymerase. Bayesian inference (BI) analysis was used to fix tree topology. BI analysis was performed on 1,000,000 iterations and nodes with a posterior probability above 0.80 are represented. ML analysis was performed on 1000 iterations and nodes above 65 are represented, when nodes coincided with BI. Recognised or a putative genera are defined as described in [27]. Substitutions models for ML and Bayesian analyses were determined as LG+I+G and rtREV+I+G, respectively. Scale bar indicates the number of amino-acid substitutions per site. Cytorhabdoviruses, Novirhabdoviruses and Nucleorhabdoviruses were excluded from the analysis because sequences were too divergent.

In order to identify whether DRV could either constitute a novel species or a novel genus within the *Rhabdoviridae* family, we selected GenBank sequences according to the Walker *et al.* dataset [27] in order to clearly identify recognised or putative *Rhabdoviridae* genera (Figure 5). Phylogenetic analysis identified a clade (sub-clade I) formed by biting midge DRV and Australian mosquito NCV, with a high bootstrap value of 99 and a high posterior probability of 1. Beaumont virus, another rhabdovirus identified in Australian mosquito metagenomes [26] and *Culex tritaeniorhynchus* rhabdovirus (CTRV), identified in Japanese mosquitoes [28] formed a sub-clade II at the root of sub-clade I (Figure 5, Figure S2). This group, consisting of the two sub-clades, could constitute a novel genus within the *Rhabdoviridae* family (Figure 5, Figure S2). This putative genus was tentatively named *Dielmovirus* genus. Dielmoviruses belong to the Dimarhabdovirus supergroup (dipteran-mammal rhabdoviruses) (Figure S2).

**Figure 6:**
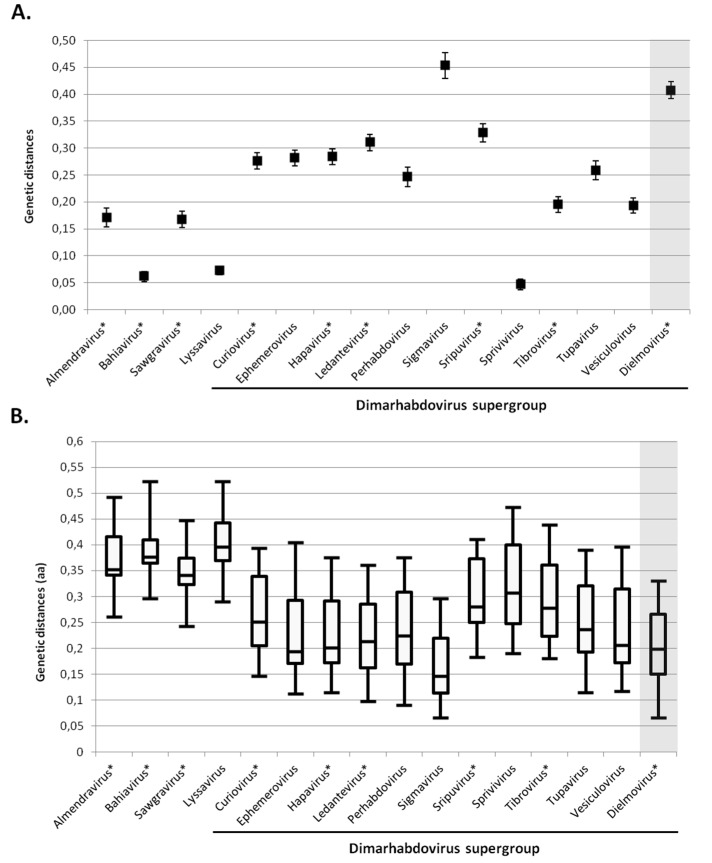
Genetic distances of *Dielmovirus* genus compared to other *Rhabdoviridae*. (**A**) Mean distances within recognised and putative *Rhabdoviridae* genera (putative genera, as reported in [27], are indicated by a *). Diversity was calculated by the pairwise-distance algorithm implemented through MEGA6 [23], and 1000 bootstrap replications; (**B**) Distribution of distances between recognised and putative *Rhabdoviridae* genera (putative genera are indicated by a *). Diversity was calculated by the pairwise-distance algorithm implemented through MEGA6 [23]

The genetic distances of *Dielmovirus* genus compared to other *Rhabdoviridae* genera, as defined by Walker *et al.* [27], are presented in Figure 6. The mean genetic distance between viruses within the *Dielmovirus* genus is higher than that observed within each recognised or putative genus (Figure 6A), with the exception of Sigmaviruses, supporting the distinction of two sub-clades within the *Dielmovirus* genus: one formed by NCV and DRV, and the other composed of Beaumont and CTR viruses. In addition, the putative *Dielmovirus* genus presented a distribution of distances with other genera in the same range than the global distribution of distances observed between other genera (Figure 6B). Viruses belonging to the *Dielmovirus* genus diverge by approximately 15%–26% in the amino-acid sequence of the RNA-dependant RNA polymerase from other *Rhabdoviridae* genera, which is globally observed for all other genera with the exception of the *Lyssavirus*, *Almendravirus*, *Bahiavirus* and *Sawgravirus* genera, which seemed to present a greater genetic distance (Figure 6B). Interestingly, these four recognised and putative genera did not belong to the Dimarhabdovirus supergroup (Figure 6B, Figure S2). The *Sigmavirus* genus presented the least distance with *Dielmovirus*, and *Bahiavirus* presented the greatest distance, which is consistent with phylogenetic observations.

### 3.5. Detection of Jingmen Tick Virus-Related Sequences

**Figure 7:**
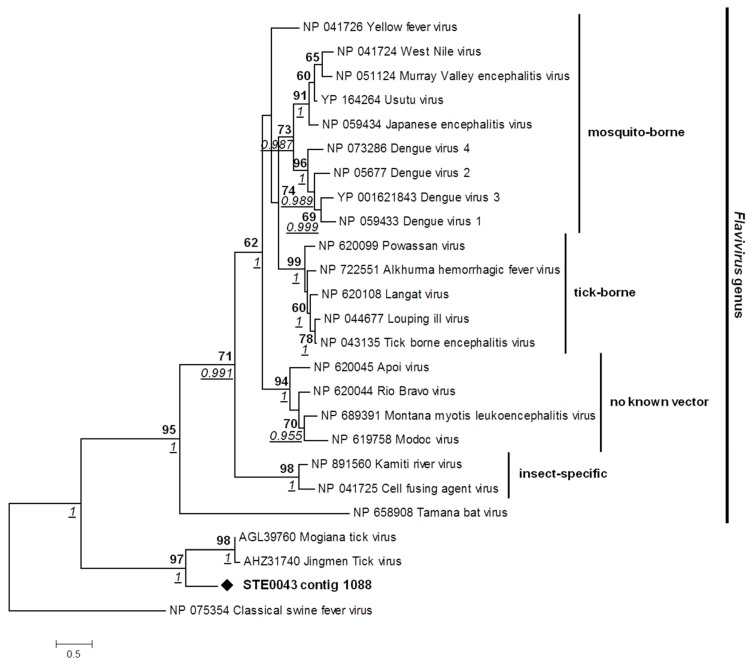
Phylogenetic analysis of Jingmen Tick-like virus. Phylogenetic analysis of a fragment of 319 amino-acids of the NS5 segment. ML analysis was used to fix tree topology. ML analysis was performed on 1000 iterations. Bootstrap values above 60 and posterior probabilities above 0.5 are indicated. Bayesian posterior probabilities are underlined and represented in italics where nodes coincided with ML. Substitution models for ML and Bayesian analyses were determined as LG+G and rtREV+ G, respectively. Scale bar indicates the number of amino-acid substitutions per site.

Within the virome of the STE0043 *Culicoides sp*. sample, one contig of 609 nt matched with the NS5 segment of Jingmen Tick virus (JTV), a novel chimerical virus isolated in Chinese ticks and composed of four segments: two originating from a flavivirus (NS3 and NS5-like segments) and two with high similarities with *Toxocara canis* nematode cDNA library [29]. The Senegalese biting midge Jingmen Tick-like virus (JTV-like virus) presented a low nucleotide identity of 57.95% with the JTV NS5 segment. Phylogenetic analysis of several representative flaviviruses, JTV and Mogiana tick virus (MTV, another virus isolated in ticks which has similarities with flaviviruses [30]) performed in the NS5 gene revealed that the Senegalese JTV-like virus was located at the root of a clade formed by these new flavi-like viruses with a high bootstrap value of 97 and a high posterior probability of 1. This clade does not belong to the *Flavivirus* genus (posterior probability of 1 for the node defining this clade apart from the *Flavivirus* genus clade), but belongs to the *Flaviviridae* family (Figure 7).

In addition, by re-analysing contigs with low identities and coverage that were previously discarded, we detected one contig which matched the JTV NS3 segment with a homology percentage of 41.3% in nucleotide and an E-value of 10^−9^, and a contig which matched *Toxocara canis* ANT-5 with an E-value of 10^−59^ and homology of 34.18%.

### 3.6. Presence of Endogenous Viral Elements?

To verify the presence of endogenous viral elements (EVE) within the major detected arboviruses, we screened for the presence of possible integration sites within the viral contigs. Among the 3′ portion of the JTV-like viral contig, only 23 nt did not match with a viral sequence but matched with *Ovis canadensis* chromosome 25. We were not able to detect similar sequences in other viral contigs.

In addition, and due to reports of a rhabdoviral EVE in mosquitoes [31], we performed a Bayesian inference phylogenetic analysis of the *Dielmovirus* rhabdovirus genus compared to other rhabdoviruses and *Rhabdoviridae*-related EVEs, which confirmed that Dielmoviruses did not correspond to the previously identified *A. aegyti* RNA-dependent RNA polymerase (RdRP)-related EVE (Figure S3).

Finally, the presence of EVEs in the glycoprotein gene of *Orthomyxoviridae* in the genome of *Ixodes scapularis* ticks had previously been reported [32], but not among the PB1 segment of the RdRP detected in our biting midge orthomyxovirus.

### 3.7. Other Viruses Present in Biting Midges

Sequencing the viral communities of Senegalese biting midges revealed the presence of viruses infecting a wide variety of hosts, including mammals, insects, plants and bacteria.

Mammalian-infecting viruses were only detected in STE0045 *C. imicola* male and non-engorged female RNA viromes and consisted of 33.73% of total viral reads (Figure 1B). The viral family which was most represented was *Picobirnaviridae* (57.59% of all mammalian viral reads). Interestingly, the *Picobirnaviridae*-related contig matched with a feline picobirnavirus with a nucleotide identity of 53.91%, suggesting the presence of a possible new picobirnavirus either originating from *Culicoides* or from animals on which arthropods feed.

Insect-specific viruses were also highly abundant in the viromes, representing 55.51%, 76.23% and 33.66% of total viral reads in the STE0043, STE0044 and STE0045 RNA viromes, respectively (Figure 1B). *Iflaviridae* were abundant, but most insect-specific viral reads matched with novel viruses, currently not recognised by the International Committee for Taxonomy of Viruses (ICTV). Indeed, sequences matching the Loreto virus, Negev virus and Negev-like virus 174, Piura virus and Nora virus were retrieved, with low nucleotide identities comprised between 50% and 56%, 50% and 60%, 63% and 74%, 51% and 63% and 67% and 69%, respectively.

Plant-infecting viruses belonging to the *Partitiviridae* and *Tymoviridae* families were detected in the STE0043 and STE0044 viromes. *Partitiviridae*-related sequences from *Alphapartitivirus*, *Betapartitivirus*, *Gammapartitivirus* genera and unclassified partitiviruses were detected in the STE0043 virome whereas only unclassified partitiviruses were identified in the STE0044 virome. All sequences displayed low nucleotide identities (53%–75%) suggesting the detection of potentially new viruses. *Tymoviridae*-related sequences, again with low nucleotide identities (51%–64%), were also detected in the STE0043 virome and were assigned to the *Maculavirus* and *Marafivirus* genera.

Several reads related to bacteriophages were also detected in the STE0044 and STE0045 samples, and amoeba-infecting giant viral sequences were identified in the STE0045 virome, probably reflecting a residual contamination of DNA in the RNA preparations (Figure 1B) or the carriage of mRNAs within viral particles.

## 4. Discussion

We report in this study an extensive characterisation of the RNA viral communities of Senegalese biting midges. Analysis of the taxonomic assignment of reads revealed a high proportion of unknown sequences. This result, in the same range as those observed in tick [10] and mosquito [8] metagenomes, again reflects the lack of data about RNA viruses in the databases, and highlights the potential pool of unknown viruses yet to be discovered and which could represent future emerging viruses.

The pattern of composition of RNA viral communities was highly divergent in terms of relative abundance and of the composition of viruses within the three metagenomes, although arthropods were collected at the same place during the same night in the same trap. This suggests that these differences may result from intrinsic characteristics of the insects rather than the environment. However, the three biting midge viromes clustered together in the principal component analysis when compared to other haematophagous and non-haematophagous arthropods, suggesting the presence of a “core” viral community shared by all biting midges, and “accessory” viral communities specific to a species, gender or haematophagous status. Indeed, STE0043 *Culicoides sp*. was more distant than the STE0045 pool of *C. imicola* males and non-engorged females and the STE0044 pool of *C. imicola* engorged females, despite the fact that they differ only by arthropod species composition. In addition, biting midge viromes were closer to other haematophagous arthropods than to non-haematophagous arthropods, potentially highlighting the influence of blood meal in the composition of viromes.

*Orbivirus*-related sequences were the most represented in the viromes. These *Reoviridae* are livestock-restricted viruses which cause significant economic losses: AHSV causes malfunctions of the circulatory and respiratory systems leading to the death of equines, while BTV and EHDV cause significant decreases in milk production and death in ruminants [15,33,34,35]. In Europe and Africa, the main vector of AHSV and BTV is *C. imicola,* while EHDV is transmitted by the *C. schultzei* group in Africa [15]. In Senegal, several *Culicoides* species are present: the *C. imicola*, *C. schultzei*, *C. milnei*, *C. magnus* and *C. fulvithorax* groups [36], which can represent a potential epizootic risk to livestock. In 2007, Senegal reported a significant AHSV epidemic among equines and, since then, animals have been vaccinated [37]. BTV also highly circulates among ruminants, as shown in sero-epidemiological studies [38], although no recent epidemics have been reported. In addition, to our knowledge, no EHD epidemic or study has been reported in Senegal, but the symptoms of BTV or EHDV infections are very similar, resulting in a possible wrong diagnosis of an etiology as a bluetongue-like pathology [15]. In this study we reported the detection of sequences related to AHSV, BTV and EHDV *Reoviridae* viruses. The STE0043 pool of *Culicoides sp*. presented the majority of *Reoviridae* reads, and within them, AHSV, BTV and EHDV represented 15.3%, 34.0% and 50.7% of total *Reoviridae*-related sequences, respectively. Interestingly, only a few BTV reads were detected in the STE0044 *C. imicola* engorged female and STE0045 *C. imicola* males and non-engorged females despite the fact that *C. imicola* is known to be the main vector of this virus in Africa. Despite the fact that this study constitutes a snapshot of the composition of viral communities present in biting midges sampled at a given time and location and may not reflect the composition of the viral communities of Senegalese *Culicoides* through one year, this result may suggest that other midge species could be vectors of BTV in Senegal, as demonstrated by the high prevalence of BTV in the STE0043 *Culicoides sp*. pool. Diarra *et al.* completed in 2014 a one-year survey of *Culicoides* midge populations in Senegal [39]. They showed that *Culicoides oxystoma*, followed by *C. kingi* and *C. imicola*, were the most prevalent species of biting midges, and they could constitute alternative vectors of BTV. In addition, the authors showed that *C. imicola* presented a globally constant abundance throughout the year lower than the ones observed for *C. oxystoma* and *C. kingi*, while *C. oxystoma* peaked during August to November and *C. kingi* during May to October, suggesting that possible other midges species may play the role of vectors of BTV in Senegal, depending on the season. Further follow-up of circulating viruses in different midge populations throughout the year would help clarify vector(s) and seasonality of BTV in Senegal. Finally, the detection of EHDV-related reads matching nearly all of the viral segments suggests that the virus is probably circulating among the vector populations and may precede the onset of an outbreak. Thus, it highlights the importance of monitoring the emergence of epizooties by studying viral communities of haematophagous arthropods [3].

In 2009, Peter Daszak noted that only 0.2% of the total estimated viral diversity possibly infecting humans is currently known [40]. By allowing the identification of potential new viruses, next-generation sequencing techniques allow reducing this gap. Indeed, in this study we reported the description of new viruses in biting midge RNA viromes, including a novel thogotovirus. Dielmo Orthomyxovirus (DOV) was detected in the pool of *Culicoides sp*. and the pool of *C. imicola* males and non-engorged females. Phylogenetic analyses and calculation of genetic distance resulted in the identification of a new thogotovirus species, distinct from other known thogotoviruses. Thogotoviruses are single-stranded RNA (ssRNA) negative-strand segmented viruses belonging to the *Orthomyxoviridae* family. All isolated from hard ticks [41] (with the exception of the Batken virus, which was also isolated from mosquitoes [42]), thogotoviruses are able to infect a wide variety of vertebrate hosts, including birds, rodents, livestock and humans [43,44,45,46,47,48,49]. In humans, these viruses cause fever and, in some cases, neurological symptoms such as meningitis or encephalitis [50,51]. Recently, a novel thogotovirus, tentatively named “Bourbon virus” was responsible for the death of an individual who had previously been bitten by a tick, due to a decrease in blood platelets and white cells but with no neurological symptoms [52]. The status of the newly described Dielmo Orthomyxovirus is currently unknown, but the successful isolation of DOV should permit to (i) determine its phylogenetic relationships with other thogotoviruses by sequencing its genome; (ii) review experiences of the vector competence of *Culicoides* midges to transmit the virus and allow its possible classification as an “arbovirus”; and (iii) develop an animal model of infection to determine its pathogenicity.

Novel *Rhabdoviridae*-related viral sequences were also detected. These sequences clustered together in a monophyletic group with North Creek virus, a virus recently discovered in *Culex sitiens* mosquitoes in Australia [26]. We propose that this sub-clade, in addition to another sub-clade formed by the Beaumont virus [26] and *Culex tritaeniorhynchus* rhabdovirus [28], form a new genus within the *Rhabdoviridae* family, tentatively named *Dielmovirus*. Dielmoviruses cluster with the *Sigmavirus* genus, within which viruses were only isolated from Drosophila flies. Many rhabdoviruses were previously isolated from biting midges [27]: for example, Fukuoka virus (a cattle virus), vesicular stomatitis New Jersey virus (a cattle virus), Wongabel virus (a seabird virus), Ngaingan virus (a cattle virus), Curionopolis virus (a primate virus) and Tibrogargan virus (a cattle virus). Nearly all of them belong to the “arbovirus” group, with the exception of the Itacaiunas virus, which is restricted to midges and which form a distinct clade. Dielmoviruses, such as the Sigmaviruses, appear to be restricted to haematophagous (mosquitoes, biting midges) and non-haematophagous (flies) Diptera, and phylogenetic analyses revealed that insect-specific rhabdoviruses form distinct monophyletic groups, suggesting that stringent host specificity occurs for these viruses (Figure 5, [27]). In contrast, arbo-rhabdoviruses, possibly due to significant host switching between vertebrate hosts and arthropod vectors, appear to be more diverse. Indeed, higher genetic distances among recognised or putative genera were observed for *Sigmavirus* and *Dielmovirus*, reinforcing the observation that strong host specificity occurs among insect-specific rhabdoviruses. Vasilakis and Tesh recently noted that insect-specific rhabdoviruses, as well as bunyaviruses and flaviviruses, are ancient and probably evolved and diversified in parallel with their insect hosts [53], via vertical transmission or integration within the host genome.

It is well-known that arthropod genomes, as well as vertebrate animals, contain integrated fragments or entire genomes of viral RNA [31,32,54,55,56,57,58]. These regions, called EVE [32], can be functional in the genomes of several hosts [58,59,60] and often derive from ancient viral infections for which the integration was vertically transmitted and evolve in parallel with their eukaryotic host. In our study, we demonstrated that the newly described *Thogotovirus* species and *Rhabdoviridae* genera did not correspond to previously reported related EVEs [31,32], suggesting that these viruses could constitute novel viral species and genus.

To conclude, this study reports the first description of viral communities of haematophagous arthropods which have an impact on human and veterinary medicine: the *Culicoides*. We detected the presence of several novel viruses, including a novel *Thogotovirus* species and a novel *Rhabdoviridae* genus, which may constitute potential risks for human and animal health. This study thus highlights the importance of characterising the viral communities of haematophagous arthropods as a first step in the evaluation of the emergence of epizooties and/or zoonoses using next-generation sequencing techniques.

## Figures and Tables

**Table 1:** Virome dataset statistics.

	STE0043 *Culicoides sp.*	STE0044 *C. imicola* Engorged ♀	STE0045 *C. imicola* Non Engorged ♂♀
Raw reads	2,071,144	1,335,388	1,507,966
Cleaned reads including:	**2,069,117**	**1,332,764**	**1,505,902**
- Paired reads	2,067,394	1,330,424	1,504,072
- Single reads	1723	2340	1830
Raw read size (nt)	301	301	301
Cleaned read size (nt)	244	244	241
Contigs	**1849**	**1139**	**1134**
Average contig length (nt)	560	536	477
Singletons	48,173	31,804	35,630
MG-RAST no	4604249.3	4604250.3	4604251.3

## Supplementary Materials

The following are available online at www.mdpi.com/1999-4915/8/3/77/s1, Figure S1: Pipeline for bioinformatic analyses, Figure S2: Phylogenetic analysis of *Dielmovirus* genus compared to other *Rhabdoviridae*. Figure S3. Phylogenetic analysis of *Dielmovirus* genus compared to other *Rhabdoviridae* and the endogenous viral element *A. aegypti*. Table S1. Characteristics of metagenomes used for PCA analysis.
