# Supplementary material for: Characterization of Viral Communities of Biting Midges and Identification of Novel Thogotovirus Species and Rhabdovirus Genus
Source: Viruses. 2016 Mar 11;8(3):77. doi: 10.3390/v8030077 (PMC4810267; doi:10.3390/v8030077)
Supplement: Supplementary file 1 [file viruses-08-00077-s001.pdf]

Article

Supplementary Material: Characterization of Viral Communities of Biting Midges and Identification of Novel *Thogotovirus* Species and *Rhabdovirus* Genus

Sarah Temmam, Sonia Monteil-Bouchard, Catherine Robert, Jean-Pierre Baudoin, Masse Sambou, Maxence Aubadie-Ladrix, Noémie Labas, Didier Raoult, Oleg Mediannikov and Christelle Desnues

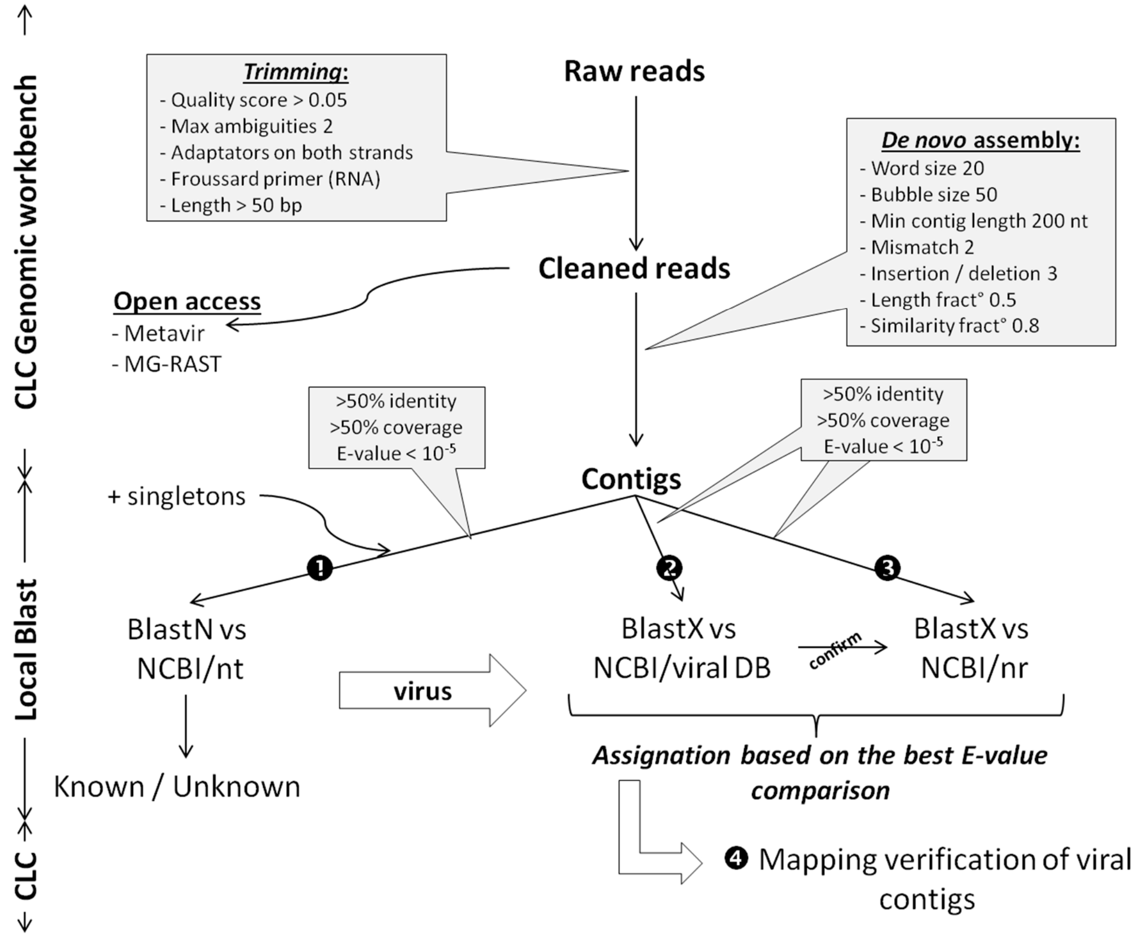

Figure S1. Pipeline for bioinformatic analyses metagenomes.

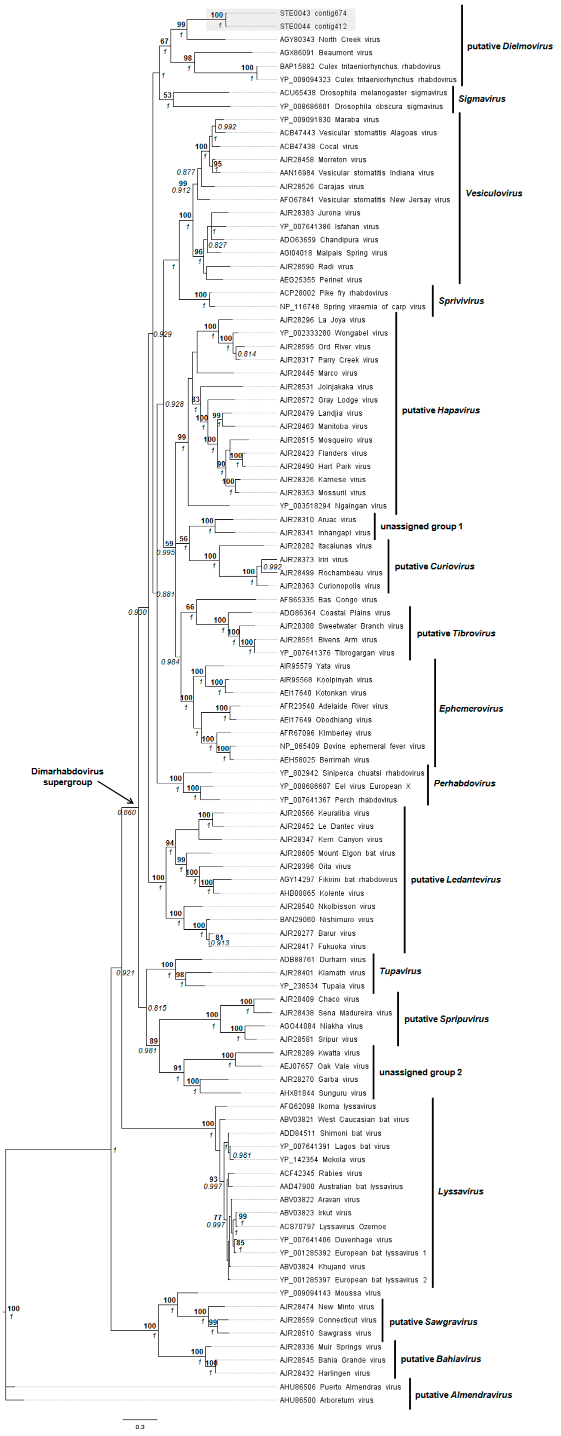

Figure S2. Phylogenetic analysis of *Dielmovirus* genus compared to other *Rhabdoviridae*. Enlarged tree of a fragment of 463 amino-acids of the RNA-dependant RNA polymerase. Bayesian inference (BI) analysis was used to fix tree topology. BI analysis was performed on 1,000,000 iterations and nodes with a posterior probability above 0.80 are represented. ML analysis was performed on 1,000 iterations and nodes above 50 are represented, when nodes coincided with BI. Recognised or putative genera are defined as described in [27]. Substitution models for ML and Bayesian analyses were determined as LG+I+G and rtREV+I+G, respectively. Scale bar indicates the number of amino-acid substitutions per site.

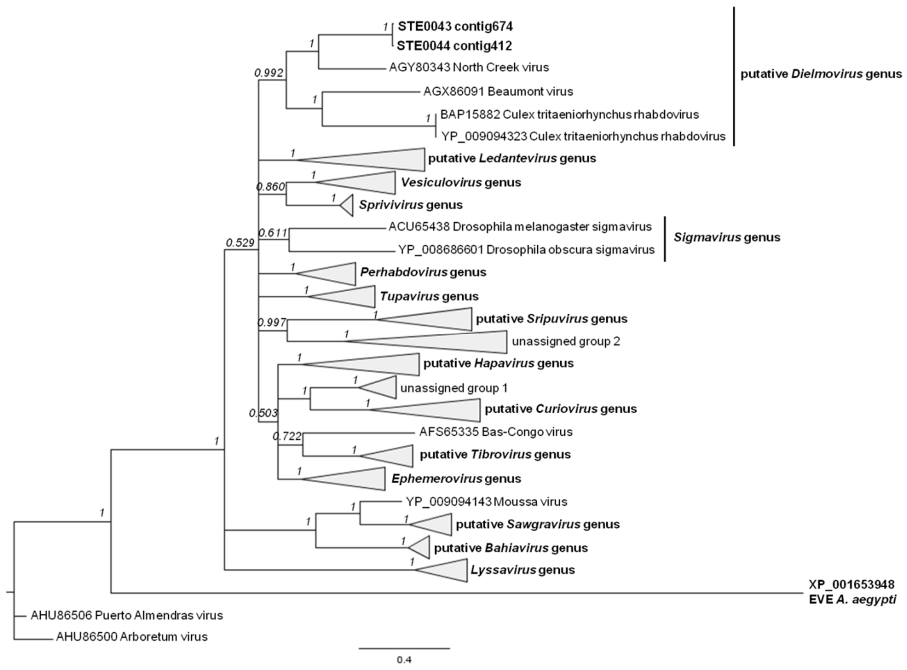

Figure S3. Phylogenetic analysis of *Dielmovirus* genus compared to other *Rhabdoviridae* and the endogenous viral element *A. aegypti*. Phylogenetic analysis of a fragment of 395 amino-acids of the RNA-dependent RNA polymerase. Bayesian inference (BI) analysis was performed on 1,000,000 iterations and nodes with a posterior probability above 0.50 are represented. Recognised or putative genera are defined as described in [27]. Substitution model was determined as rtREV+I+G. Scale bar indicates the number of amino-acid substitutions per site.

Table S1. Characteristics of metagenomes used for PCA analysis**.**

Arthropod metagenome	Haematophagous	Base no	Reads no	Sequencing method	Remarks	Ref.
**Mosquitoes**	yes	68,708,092	289,436	Roche 454 FLX	artificially infected with DENV-1	4
		49,582,727	216,164	Roche 454 FLX		
		53,556,733	341,650	Roche 454 FLX		
		78,813,957	390,971	Roche 454 FLX		
		67,224,921	336,822	Roche 454 FLX		
**Mosquitoes**	yes	16,431,897	89,744	Illumina GA II	-	6
**Mosquitoes**	yes	20,087,132	29,234	Illumina HiSeq 2000	assembled dataset	7
		32,513,784	44,558	Illumina HiSeq 2000		
		79,323,266	110,242	Illumina HiSeq 2000		
		44,945,652	53,542	Illumina HiSeq 2000		
		20,382,748	29,911	Illumina HiSeq 2000		
		18,912,844	13,577	Illumina HiSeq 2000		
		21,145,145	30,686	Illumina HiSeq 2000		
**Whiteflies**	no	63,168,431	1,427,809	Illumina GA II	-	61
**Butterflies**	no	37,301,814	82,099	Sanger	-	62

© 2016 by the authors; licensee MDPI, Basel, Switzerland. This article is an open access article distributed under the terms and conditions of the Creative Commons by Attribution (CC-BY) license (http://creativecommons.org/licenses/by/4.0/).
